# Application-Oriented Retinal Image Models for Computer Vision

**DOI:** 10.3390/s20133746

**Published:** 2020-07-04

**Authors:** Ewerton Silva, Ricardo da S. Torres, Allan Pinto, Lin Tzy Li, José Eduardo S. Vianna, Rodolfo Azevedo, Siome Goldenstein

**Affiliations:** 1 Institute of Computing, University of Campinas, Campinas 13083-852, Brazil; ewerton.silva@students.ic.unicamp.br (E.S.); allan.pinto@ic.unicamp.br (A.P.); lintzyli@gmail.com (L.T.L.); jevianna@gmail.com (J.E.S.V.); rodolfo@ic.unicamp.br (R.A.); siome@ic.unicamp.br (S.G.); 2 Department of ICT and Natural Sciences, Norwegian University of Science and Technology, Ålesund, 2 6009 Larsgårdsvegen, Norway

**Keywords:** retinal image model, space-variant computer vision, foveation, low-power, energy consumption

## Abstract

Energy and storage restrictions are relevant variables that software applications should be concerned about when running in low-power environments. In particular, computer vision (CV) applications exemplify well that concern, since conventional uniform image sensors typically capture large amounts of data to be further handled by the appropriate CV algorithms. Moreover, much of the acquired data are often redundant and outside of the application’s interest, which leads to unnecessary processing and energy spending. In the literature, techniques for sensing and re-sampling images in non-uniform fashions have emerged to cope with these problems. In this study, we propose Application-Oriented Retinal Image Models that define a space-variant configuration of uniform images and contemplate requirements of energy consumption and storage footprints for CV applications. We hypothesize that our models might decrease energy consumption in CV tasks. Moreover, we show how to create the models and validate their use in a face detection/recognition application, evidencing the compromise between storage, energy, and accuracy.

## 1. Introduction

By means of a conventional sensor, one can easily capture uniform high-resolution images and describe what is depicted. However, for computers, interpreting images is not trivial, demanding complex Computer Vision (CV) algorithms, along with a proper management of the available resources, to allow the software applications to run efficiently in different hardware platforms. As a matter of fact, a computational burden might come into play due to real-time restrictions that are often imposed by the available hardware to process these high-resolution data [1]. In the mobile environment, for example, managing energy (i.e., battery life) is mandatory, as its negligence might prevent users from enjoying a satisfactory experience [2]. Whereas common strategies to save resources rely on uniform resolution reductions and frame-rate decreases, another one is to mimic the space-variant configuration of the human eye. Because some tasks as tracking and pattern recognition do not demand high resolution data across the whole image [1], it is reasonable to work with space-variant images.

The paradigm of capturing and processing uniform images co-exists with mechanisms to manage a biology-inspired image representation in the Space-Variant CV field. The overall insight comes from the nature of the human eye, where cones and rods—the photo-receptors that are responsible for detecting color and luminance, respectively—show a non-uniform spatial configuration that induces variable visual acuity levels across the retina [3]. The highest density of cones lies in the fovea, the central area of the retina, whereas the lowest one is found across the periphery. This provides a wide field of view and a high-resolution region that is used to *foveate* a point in a real scene, thereby reducing data processing to a dense, smaller region (fovea), or to a wider, sparse one (periphery) [3,4]. Both regions can also operate in synergy: the periphery examines coarse data in order to trigger a detailed analysis through foveation.

Despite the progress in CV research fields in exploiting space-varying models, there is a lack of a single generic framework for handling seamlessly images generated by heterogeneous pixel sampling strategies. In this paper, we address this issue by proposing a framework for designing Application-Oriented Retinal Image Models (ARIM) that establish a non-uniform sampling configuration of uniform images. We propose defining the appropriate model for an application on-demand, while taking specific requirements of the target application into account. By exploiting such models, we hypothesize that it might be possible to decrease the energy spent in computer vision tasks. We show how to create the models and validate their use in a face detection/recognition application, when considering the compromise among storage rates, energy, and accuracy. We use a regular image sensor and perform the sampling procedures by means of a software layer, thus simulating the operation of a specific-purpose space-variant sensor and providing some flexibility. Our main contributions are the following:

- we provide a framework for designing Application-Oriented Retinal Image Models (ARIM) towards computer vision (CV) applications;
- we evaluate the use of ARIMs in a CV application of the biometry field in terms of memory storage and energy reductions;
- we discuss the trade-offs between the application’s accuracy and the reductions in the computing resources induced by the ARIMs;
- we compare our results to other common setups (original and downsized uniform-resolution images) and show that the obtained storage and energy savings are relevant; and,
- we briefly discuss the use of ARIMs in real-life application scenarios and the nuances of having an ideal hardware layer that resamples images according to ARIMs.

The paper is organized, as follows. We conduct a literature review on the subject in Section 2. In Section 3, we detail the proposed framework to generate ARIMs. In Section 4, we present information regarding the experimental protocol for simulating the use of ARIMs, such as the employed dataset, the application implementation, the evaluated models, and the evaluation criteria, along with the adopted hardware setup. We present experimental results regarding storage, accuracy, and energy in Section 5; in this section, we also discuss these results and show their implications in real-life application scenarios. We conclude our work and describe the possibilities of future studies in Section 6.

## 2. Literature Review

Concepts of the human visual system have already been explored from the hardware and software perspectives. In this section, we review some of the works from each perspective. A more complete review on space-variant imaging from the hardware and software perspectives while using log-polar mappings is detailed in [5]. We also describe some works regarding recent clustering techniques. Although we are not tackling the challenges of data clustering during the generation of ARIMs, the idea of obtaining a low-dimensional representation for the points (extracted from face images) could further reduce the memory storage use of the CV application.

### 2.1. Hardware-Based Approaches

On the hardware side, the problem has been dealt with, mainly, by two fronts: (i) the development of imaging sensors with specific non-uniform spatial configurations [6] and (ii) the use of an intermediary hardware layer to remap uniform images into variable-resolution ones. The first front allows to capture topology-fixed foveated images at sensing time, whereas the second one provides more flexibility to change the mapping without relying on software routines. Specifically, some initiatives, like [1], exploited the versatility of Field Programmable Gate Arrays (FPGA) to implement, at a logical level, different space-variant mappings of uniform images, as with the case of a moving fovea that is dynamically adjusted according to the application’s requirements.

A similar study [7] integrated attention and segmentation mechanisms into a foveal vision system. The architecture of the solution comprised (i) a hardware layer responsible for mapping uniform cartesian images to space-variant ones and (ii) a software layer where segmentation and saliency estimation are done. In short, the salient regions from a frame might trigger a foveal shift to be performed by hardware when the next frame arrives.

### 2.2. Software-Based Approaches

Pure software-based approaches, in opposition to hardware-oriented ones, offer more flexibility to simulations, albeit with higher computational costs. In [8], a saccadic search strategy that is based on foveation for facial landmark detection and authentication is presented. The authors apply a log-polar mapping to some image points and extract Gabor filter responses at these locations, thus imitating the characteristics of the human retina. For training, Support Vector Machine classifiers are used to discriminate between positive and negative classes of facial landmarks (eyes and mouth) represented by the collected Gabor responses. When testing, the saccadic search procedure evaluates several image points in the search of candidate landmarks that are further used to authenticate the depicted individual.

A foveated object detector is proposed in [9]. The detector operates on variable-resolution images obtained by resampling uniform ones with a simplified model of the human visual cortex. The results showed that the detector was capable of approximating the accuracy of a uniform-resolution-oriented one, thereby providing satisfactory insight to evolutionary biology processes.

In another work [10], image foveation is exploited along with a single-pixel camera architecture to induce a compromise between resolution and frame rate. The images are resampled by a space-variant model that is constantly reshaped to match the regions of interest detected in the image by a motion tracking procedure, thus effectively simulating a moving fovea that increasingly gathers high-resolution data across frames.

An appropriate method is described in [11] to facilitate comparisons among different sensor arrangements. The idea is to provide a common space for creating lattices of any kind. To demonstrate the viability of the method, the rectangular and hexagonal lattices are implemented and images built according to both arrangements are further compared.

### 2.3. Recent Clustering Techniques

As a very popular unsupervised learning approach, clustering has been deeply explored by researchers and used in several contexts. This technique aims to partition a set of data into groups where the elements share similar properties [12]. For instance, clustering is useful to group unlabeled images (represented by feature vectors) according to some desired criteria. In fact, many CV applications employ clustering at some point of their architectural design, such as content-based image retrieval systems [13] and face image clustering [14].

Recently, deep learning-based approaches have arisen to improve the results of clustering procedures. In [15], a deep subspace clustering technique is proposed. The core of the approach resides in mapping the data points into latent subspaces and preserving local and global structural properties simultaneously. The method also provides good scalability, since the nonlinear mapping is progressively done for the input dataset. The method is shown to perform better than many state-of-the-art subspace clustering techniques in terms of clustering quality, but also presents higher execution times as compared to the evaluated literature.

In [16], a novel clustering method is proposed. The method’s central idea is based on the observation that a metric-invariant space can be learned, thus leading to the invariant sample assignment property of the clustering. Such a property is of much relevance, because it allows for distinct distance metrics to map a sample point to similar (sometimes even to equal) clusters. A deep neural network is employed to learn a better representation for the points, so that the clustering accuracy and discrepancy between clusters are satisfactory. The method works, even in the scenario where no manual annotation is provided and generates good clustering results due to the better representations found for samples.

Inspired by the ideas of these previous initiatives, we elaborated a framework to design ARIMs. The models represent the expected configuration of space-variant images, as defined by a prior analysis of the CV application’s domain. As we will show in the next section, an image resampled by using an ARIM presents variable resolutions across its space. These areas may be exploited in different ways in order to save computing resources and still allow for satisfactory accuracy rates for the desired application.

## 3. Proposed Approach

**Figure 1:**
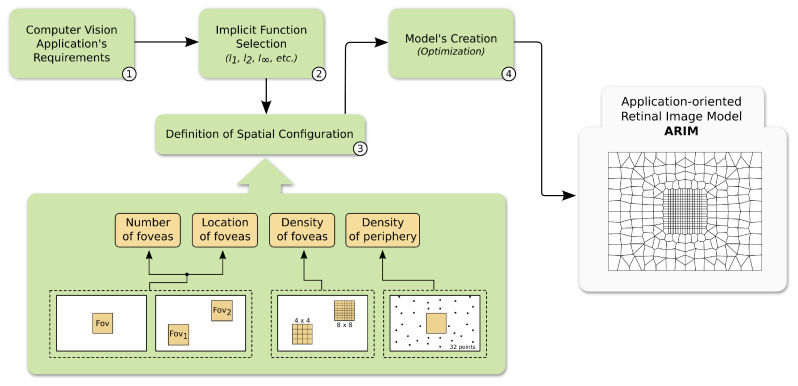
The proposed framework to generate application-oriented retinal image models (ARIMs). The workflow begins by defining the application’s requirements regarding operation (e.g., objects’ positioning, illumination) and efficiency (e.g., storage, accuracy). Then, a proper implicit function (e.g., $l_{2}$) and the spatial configuration of the retinal image model—comprising foveal and peripheral regions—are chosen. The next step is the generation of the model by means of an optimization procedure that considers the implicit function and the spatial configuration to resample points in the 2D cartesian space. The final artifact is an ARIM comprised by uniformly- and non-uniformly-sampled foveal and peripheral regions, respectively. This model is used to resample uniform images, taking them to a space-variant domain and potentially contemplating the requirements determined beforehand.

In this section, we describe our methodology to generate ARIMs. Figure 1 shows the steps of the proposed framework, which we detail in this section. The components of the proposed methodology will be presented in the context of a biometric application.

### 3.1. Definition of Application Requirements

Instead of using a traditional image, coming from a general uniform sensor, we argue that the best approach is to examine the target application and investigate its requirements and demands. CV applications can comprise a very diverse set of requirements, ranging from efficiency-related ones, such as storage, speed, energy, and accuracy, to other very application-specific ones, such as the need for objects to move slowly or be positioned in specific locations in the scene, be situated in a minimum/maximum distance from the camera, be illuminated by a close light source, and so further. The application considered in this paper is concerned with user authentication based on his/her face: the individual enters and leaves the scene by any sides, placing himself in front of a camera that captures the scene in a wide field of view.

Although the authentication across a wide field of view is a good idea, because more faces are collected throughout the video, it is usual that the central part of the image be the protagonist of the process. In this vein, it is recommended that the individual stands or walks near the center of the image to proper positioning his/her face (e.g., to avoid severe rotations and perspective changes) for a more accurate authentication process. Thus, if one intends to reduce energy consumption, collecting faces only in a bounded central region (e.g., a square window) might be enough. On the other hand, restricting the image to its central part, albeit effective, might be seen as a very extreme decision, since other image areas may contribute with useful information for the authentication. In this sense, retaining some pixel data in such areas, even in a sparse manner, is also appropriate. Finally, another suitable strategy towards energy reduction is downsampling the image before performing face detection/recognition. This might reduce the energy that is spent in the whole authentication process, but at the cost of a drop in accuracy.

The issues that are discussed above illustrate examples of requirements to be defined by the analysis of an application’s domain. In this paper, they were essential to guide the definition of a model for the biometric application.

### 3.2. Implicit Function Selection

**Figure 2:**
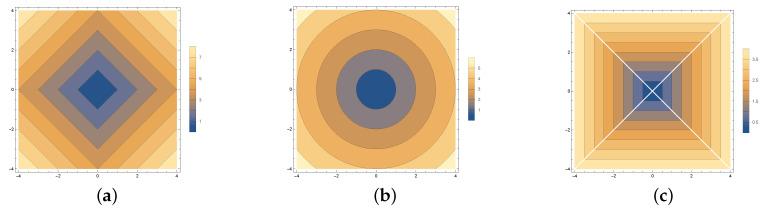
Examples of implicit functions: (**a**) $l_{1}$, (**b**) $l_{2}$, and (**c**) $l_{\infty }$.

The design of the model starts with selecting a proper implicit function. The idea is that the function will act as a control mechanism to spread out the non-uniform sampled points over a desired image region. Figure 2 depicts examples of implicit functions that we explored ($l_{1}$, $l_{2}$, and $l_{\infty }$).

### 3.3. Definition of Spatial Configuration

This step is concerned with the spatial characteristics that the model must obey. We developed hybrid space-variant models inspired on the human retina. In general, the models comprise two very distinct regions: the fovea and the periphery. The fovea is a fixed-size region of uniformly sampled pixels according to a predefined grid. For instance, a region of size $2^{6}\times 2^{6}$ pixels can be uniformly sampled by a grid of size $2^{5}\times 2^{5}$ pixels. Given these characteristics, we can apply conventional CV algorithms in the fovea. In opposition, the periphery is a fovea-surrounding region with a non-uniform pixel density that decreases with the distance from the fovea.

The following four parameters should be informed prior to the creation of the hybrid model:

- Number of foveas: surely a human eye has only one fovea, but it is perfectly fine for a model to comprise more than one region of uniform sampling, depending on the application on hand. In our biometric application, we took into account only one fovea.
- Location of foveas: the foveas should be spatially organized adhering to the specific requirements of the application. In ours, the fovea is centralized in the image.
- Density of foveas: the foveas can be downsampled to simulate a uniform image resolution reduction. We tested different densities (grids) for our fovea.
- Density of periphery: the periphery is an important region that encompasses few sparse data in a non-uniform sampling configuration. As discussed previously, by retaining and wisely handling sparse peripheral information (e.g., detecting motion and coarse objects in such an area), the application’s resource usage might be optimized.

### 3.4. Model Generation

There are several ways to achieve a non-uniform point distribution. Our approach is inspired by the computer graphics literature and previous works [17,18]. Besides the implicit function, the number of peripheral (non-uniform) points and the aspect ratio of the sensor must be provided. We generate a point distribution via a local non-linear optimization procedure that, from an initial distribution, tries to minimize a global energy function that is defined in Equation (1), where $\vec{x}$ is a point in image space.

(1) $$En\left(\{{\vec{x}}_{i}\}\right)=\sum_{i}\sum_{{\vec{x}}_{j}↔{\vec{x}}_{i}}\left(\left|\right|{\vec{x}}_{i}-{\vec{x}}_{j}\left|\right|-\left(f\left({\vec{x}}_{i}\right)+f\left({\vec{x}}_{j}\right)\right)\right)$$

**Figure 3:**
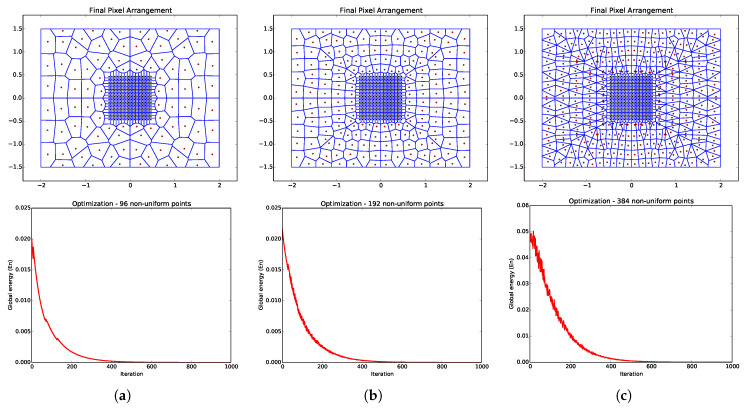
Convergence analysis for ARIMs with a single fovea and based on the $l_{\infty }$ implicit function. Examples of ARIMs containing (**a**) 96, (**b**) 192, and (**c**) 384 non-uniform points in the periphery. For each ARIM, the resulting global energy curve over 1000 iterations of the generation process is shown in the model’s respective column.

**Figure 4:**
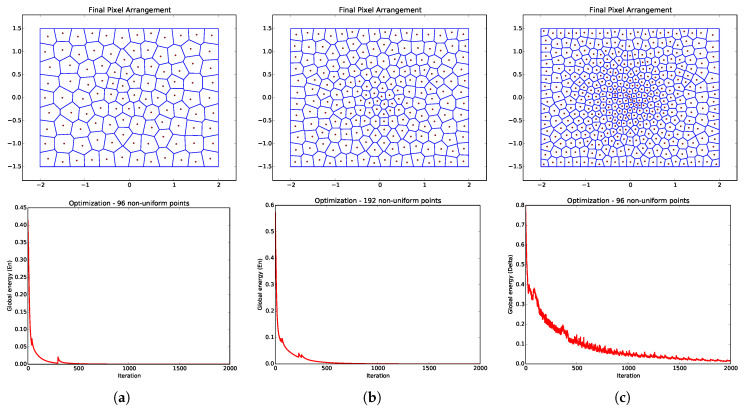
Convergence analysis for ARIMs with no fovea and based on the $l_{2}$ implicit function. Examples of ARIMs containing (**a**) 96, (**b**) 192, and (**c**) 384 non-uniform points in the periphery. For each ARIM, the resulting global energy curve over 2000 iterations of the generation process is shown in the model’s respective column.

The optimum solution for Equation (1), i.e., when En=0, would be a placement of every ${\vec{x}}_{i}$, such that the distance to its “neighbors” is the sum of the values of the implicit function at their locations. However, there is no closed-solution for this problem (the implicit function can be anything) or any guarantees of a perfect solution for a scenario with an arbitrary number of points and implicit functions. Thus, we propose an approximation by means of a non-linear optimization procedure based on Mass-Spring Models. When doing so, each pair of points tries to attract each other if they are too far, and try to repel each other when they are too close. We do not use Newton’s physical model of forces from springs. Instead, we have a mass-free system, so springs generate “velocity forces.” Figure 3 and Figure 4 show the behavior of the global energy optimization for models with different configurations, implicit functions, and number of points.

**Figure 5:**
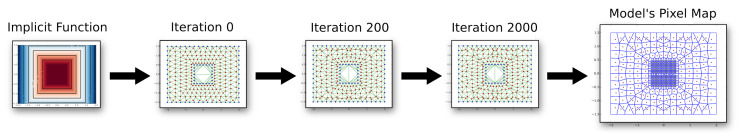
The evolution of an example of ARIM with 256 foveal (uniform), and 192 peripheral (non-uniform) pixels. The $l_{\infty }$ is the implicit function.

The optimization process is very sensitive to its initial conditions. A uniform distribution of the initial positions over the valid domain coupled with a careful choice of the implicit function allows for the system to converge under 2000 iterations. Figure 5 illustrates the generation of an ARIM, where the optimization of uniform point distribution is carried out using the $l_{\infty }$ implicit function. Upon convergence, we obtain the full neighborhood map (Voronoi diagram) of the model.

## 4. Materials and Methods

In this section, we present the experimental setup that is necessary for simulating the usage of the proposed models. The chosen dataset closely resembles one of a biometric application.

### 4.1. Target Application: Face Detection/Recognition

In this paper, we selected a CV application from the biometry domain to evaluate the proposed framework.

#### 4.1.1. On the Application Selection

Although the framework may be appropriated to many CV applications, such as those that are related to surveillance and remote sensing, the biometry domain is characterized by well-consolidated techniques and datasets, due to the several studies in the area over the years. Moreover, the considered application could fit the complete process that is described in Section 3. In this case, the application’s characteristics and requirements could be examined by reasoning about each step of the framework (Figure 1), which we report herein:

- Step 1: We analysed the CV application’s demands and characteristics. In the biometry application considered, we observed aspects regarding:
  1. the use of computational resources, which should be preferably low when running in environments of large energy and storage limitations;
  2. the intrinsic characteristics of the application’s domain, such as the task to be executed (face authentication), the expected “behavior” of the input data (person movement and positioning in the images), camera angles, the most relevant part of the image to process, etc.;
  3. the possibility of balancing the pixel density of different image regions. In this sense, given the application on hand, we decided that the processed image would have different resolutions across its space. This will induce a compromise between energy, storage, and accuracy; and,
  4. the possibility of adopting distinct pixel representations across the image in order to save computational resources. In the current case, an additional motion analysis is performed by taking advantage of an optical flow pixel representation in some image regions.
- Step 2: subsequently, by the previous analysis, we selected an appropriate implicit function to represent the pixel distribution of the image;
- Step 3: next, we defined the spatial configuration of foveal and peripheral regions by knowing, for instance, that individuals often move to the central part of the image to allow a better authentication. In this case, we defined a single central fovea;
- Step 4: finally, we created ARIMs encompassing and consolidating the expected properties of the images defined in the previous steps.

#### 4.1.2. On the Application Implementation

The face recognition process is based on the classical nearest neighbor strategy. In the training stage, we took the P1E_S1_C1 sequence and extracted the faces of all 25 individuals. We chose that sequence because the camera angle favored the position of faces. Then, we created a face training dataset consisting of 25 classes, each one having five samples. To train the faces, the DNN model extracted the 128-d features from all samples. In the test stage, we extracted the features from an unknown detected face and compared it to all the others from the training dataset using the Euclidean distance metric. The label of the closest face was considered as the label for the unknown face, thus resembling a 1-Nearest Neighbor (1-NN) strategy.

#### 4.1.3. Simulation Details

We simulated the operation of a specific-purpose sensor by re-sampling images according to our ARIMs. The idea was to generate images containing two regions: (i) the fovea, encompassing a small area where resolution is uniform, and (ii) the periphery, where pixels are arranged non-uniformly over a wider area. With such a configuration, we were able to perform experiments when considering different foveal resolutions, while also taking advantage of the periphery according to the specific requirements of the application. In this sense, we adopted an optical flow representation (orientation and magnitude) for peripheral pixels. The motivation around that representation is that the detection/recognition in the fovea could only be triggered when there is movement towards it coming from the periphery. Additionally, both the detection and recognition procedures turn off when no face is found under a predefined time interval. Therefore, in this scenario, more energy can be saved.

**Figure 6:**
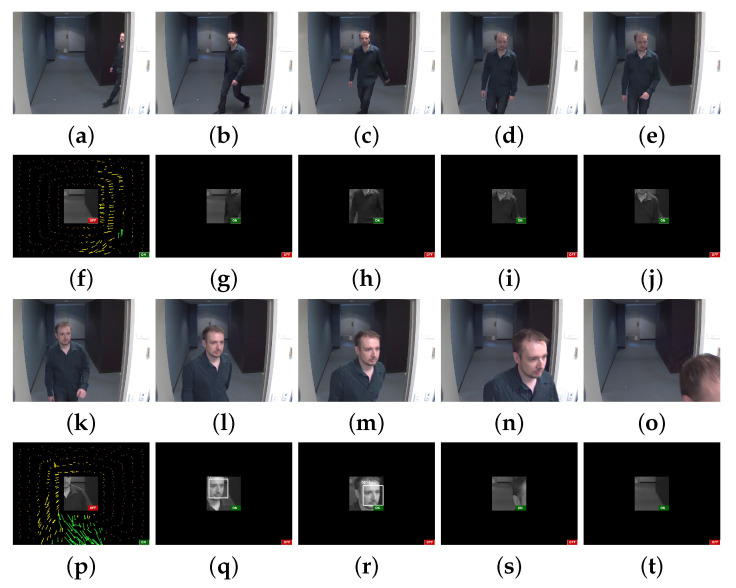
Example of a simulation using one of our ARIMs and a sample sequence from the employed dataset [19]. Images (**a**–**e**) and (**k**–**o**) are the original frames; images (**f**–**j**) and (**p**–**t**) are the reconstructions with a model that considers an optical flow peripheral representation. Green and yellow arrows indicate motion direction to the right and left sides, respectively, whereas the ON and OFF labels refer to the operational status of the foveal (face detection/recognition) and peripheral (optical flow) regions. Note that the motion analysis, besides triggering foveal analysis, is also able to restart conveniently, as long as faces are not detected in the fovea during a time interval of frames (left-most frame in the fourth row).

Figure 6 shows an example of a simulation using one of our ARIMs and a sample sequence from the employed dataset [19]. The first and third rows show the original frames, while the second and fourth rows show images reconstructed with a model that considers an optical flow peripheral representation. Green and yellow arrows indicate motion direction to the right and left sides, respectively, whereas the ON and OFF labels refer to the operational status of the foveal (face detection/recognition) and peripheral (optical flow) regions. Besides triggering foveal analysis, the motion analysis is also able to restart conveniently, as long as faces are not detected in the fovea during a time interval of frames (see Figure 6p).

Ideally, an ARIM should be first computed by software in an offline step. Subsequently, at the application’s run-time, the computed model should be an input to a reconfigurable hardware layer that will extract the necessary pixel information from a full-size image captured by a conventional uniform image sensor. Additionally, optical flow should be computed only for the peripheral points, thereby further discarding more image data. As foveal and peripheral pixel configurations do not change often for a specific application, the computational cost to control this discarding procedure by hardware should be low.

**Figure 7:**
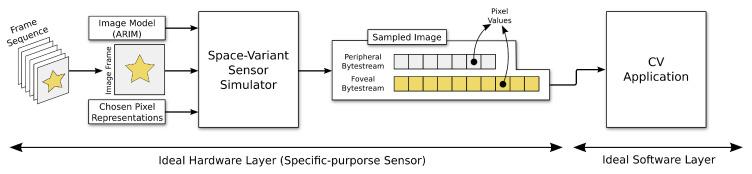
Implemented workflow for simulating the use of ARIMs in a specific Computer Vision (CV) application. In an ideal scenario, the ARIM, a captured image frame, and the chosen pixel representations for foveal and periphery areas are input to an hypothetical specific-purpose sensor that changes its configuration at run-time. Such a sensor would yield a stream (bytestream) of pixel data from each region of the captured image. The stream (not the 2D image) would be forwarded to the CV application. However, for simulation purposes, this architecture is fully implemented by software.

The workflow of the simulation process is depicted in Figure 7, where we distinguish between the software and hardware layers to illustrate a hypothetical case where a specific-purpose (space-variant) sensor was available. In an ideal scenario, the ARIM, a captured image frame, and the chosen pixel representations for foveal and periphery areas are input to an hypothetical specific-purpose sensor that changes its configuration at run-time. Both layers are connected by a 1-d vector (named as bytestream) that stores the foveal and peripheral pixel values captured by the sensor (i.e., the sampled image), and are input to the application. For simulation purposes, however, this architecture is fully implemented by software. We adopted bytestreams instead of a two-dimensional (2D) image representation in the software simulation to bring the process closer to the ideal conceived scenario. The simulator was implemented in C++ using the OpenCV 3.0.0 library.

#### 4.1.4. Technical Information

The biometric application uses the Viola–Jones [20] algorithm, which is a well-consolidated and widely used face detection method in the literature. As for recognizing faces, we used a descriptor based on a pretrained Deep Neural Network (DNN) model, which is essentially a ResNet network with 29 convolutional layers (based on [21]) trained on a dataset containing approximately 3 million faces. The model is publicly available and integrates the Dlib C++ Library [22]. These and other technical information, such as the methods and parameters used, are displayed in Table 1.

### 4.2. Dataset

In our evaluations, we employed the ChokePoint dataset [19] aimed at person identification/verification. The dataset comprises 48 sequences of images of 800×600 pixels resolution and is publicly available. Each sequence depicts several individuals entering or leaving a portal, one at a time. There are 25 and 29 individuals walking through portals 1 and 2, respectively. Moreover, each sequence is registered by three cameras that are placed above the portals to provide diverse sets of faces in different illumination and pose conditions. Due to the adopted settings, one of the cameras is able to capture image sequences of near-frontal faces. Figure 6 shows a sample sequence from the employed dataset in the first and third rows.

#### 4.2.1. Justification for the Selected Dataset

The dataset was chosen because it represents a satisfactory scenario where a CV biometry application may take place via a detailed investigation of its characteristics and requirements. When an individual is about to cross the portal, his/her face gets well centered in the image, providing the application with the necessary data to perform the biometry procedures. Furthermore, individuals do not suddenly appear in the center of the images; they slowly move towards the portal. This peripheral movement data could be exploited to activate the authentication in the central region of the image (where a face is supposed to be).

#### 4.2.2. Dataset Organization

The dataset is partitioned into the following four subsets:

- P1E and P1L: the subsets of frame sequences of people entering and leaving portal 1, respectively;
- P2E and P2L: the subsets of frame sequences of people entering and leaving portal 2, respectively.

A subset is comprised of four (4) frame sequences (S1, S2, S3, and S4), each of which is registered by three cameras (C1, C2, and C3). For instance, the frame sequence P1E_S2_C3 refers to the second sequence (S2) of people entering portal 1 (P1E) and captured by camera 3 (C3).

We used 34 image sequences (out of 48) from the dataset during our evaluations due to the following reasons:

1. One (1) of the sequences of individuals entering a portal (P1E_S1_C1) was used to train the face recognizer. Such sequence comes from camera 1, which obtains near frontal-face images. That sequence is also captured by cameras 2 and 3 at different angles, hence, to avoid biased evaluations, we ignored such sequences (P1E_S1_C2 and P1E_S1_C3), as both of these contain, essentially, the same faces of the former up to slight angle variations.
2. Eleven (11) sequences where no face is found in the fovea were ignored. This decision was taken because no face recognition accuracy evaluations (using our models) would apply to these sequences.

### 4.3. Evaluated Models

**Figure 8:**
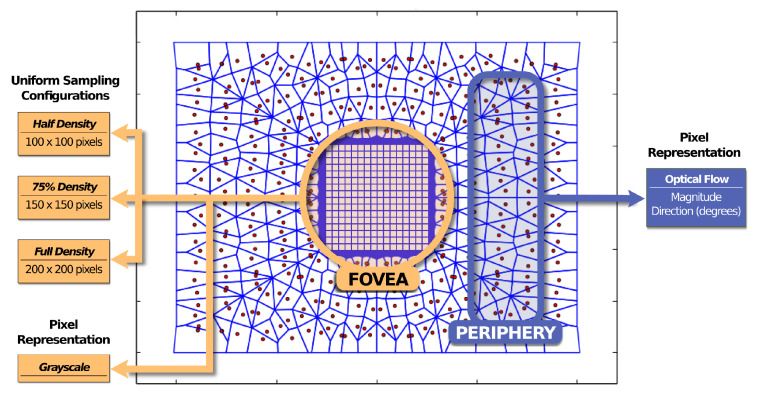
The pixel map of the evaluated ARIM and its configurations. The experimented foveal configurations comprised three uniform sampling setups: 100×100 (half density), 150×150, and 200×200 (full density) pixels. The pixel representations for the fovea and periphery were based on the grayscale and optical flow (magnitude and direction) values, respectively.

We evaluated three different ARIMs. Each model comprises 384 non-uniform peripheral points and a central foveal region of size 200×200 pixels. The models diverge from each other in the uniform-sampling configuration sizes adopted for their foveas, which are 100×100 (half density), 150×150 (75% density), and 200×200 (full density). These settings allow for us to simulate different foveal resolutions. For all models, optical flow peripheral information is used to trigger the face detection/recognition in the fovea. Figure 8 shows an illustration of the pixel map of these models and their configurations.

### 4.4. Evaluation Criteria and Hardware Setup

We compared the storage usage by computing the amount of bytes for storing the video, measured the energy spent (in Joules) in the biometric application for each evaluated model, and computed the mean recognition accuracy of each evaluated model when considering all video frames. To measure energy, we used the Intel RAPL (Running Average Power Limit) interface [24], which is a set of internal registers from Intel processors, called model specific registers (MSR). At the code level, we read these registers before and after a block of instructions, and calculate the difference between these values. More specifically, we read the MSR_RAPL_POWER_UNIT register to measure the energy spent in image readings, face detection/recognition procedures, and optical flow analysis (when using ARIMs). The hardware setup to perform the experiments comprised an Intel Core i7-5500U, with 2.04 GHz clock, 4 MB cache, and 16 GB RAM.

## 5. Results and Discussion

In this section, we present the experimental results regarding storage allocated, face recognition accuracy, and energy consumption induced by different ARIMs. We also discuss these results and their implications for real-time applications.

### 5.1. Storage Reduction

Quantifying reductions in the numbers of pixels and image data sizes is essential for assessing the benefits of using different ARIMs in practical situations. Table 2 shows these measurements for the original (full-size) images, images uniformly resized, and the three evaluated models. When compared to original images, ARIMs showed a reduction of more than 91% in the number of pixels and bytes, whereas a uniform resize of the images to 25% of their original sizes provided a reduction of 75% in both quantities.

### 5.2. Face Recognition Accuracy

We defined accuracy as the number of true positives (i.e., correctly labeled faces) in the foveal region of a frame sequence, each of which has a benchmark for comparison. The employed dataset informs all faces that appear in each image frame. However, for a fair accuracy comparison among the uniform images and the ones re-sampled by our models, we only use the information regarding the foveal region as benchmark, meaning that faces in the periphery are not considered.

**Figure 9:**
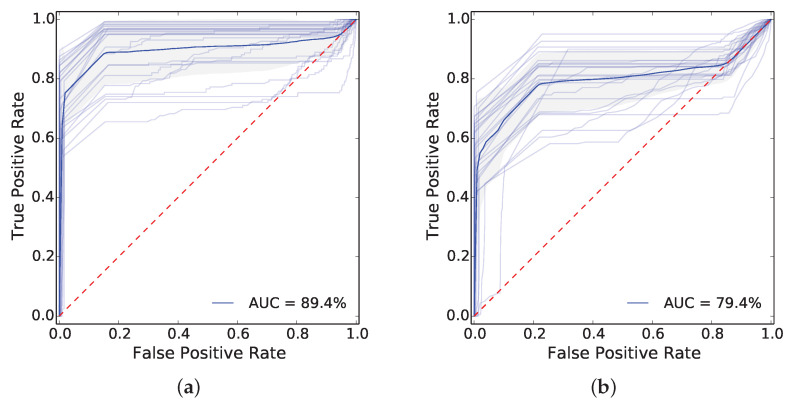
ROC curves regarding the face recognition task considering the (**a**) P1E_S2 and (**b**) P1L_S2 image sequences from all cameras. The figures comprise a mean ROC (blue) curve from all 25 (light blue) class-specific curves, i.e., for each face class in the dataset. These class-specific ROC curves were calculated via a one-versus-all classification procedure.

First, our face recognizer alone has satisfactory accuracy. Figure 9 shows receiving operating characteristic (ROC) curves regarding the face recognition task when considering the P1E_S2 (Figure 9a) and P1L_S2 (Figure 9b) image sequences from all cameras. The figures comprise a mean ROC (blue) curve from all 25 (light blue) class-specific curves, i.e., for each face class in the dataset. These class-specific ROC curves were calculated via a one-versus-all classification procedure and they evaluate the accuracy of our classifier in the experimented dataset (i.e., full-size images). We did not consider any models to generate the curves. The area under the curve (AUC) is greater for the P1E_S2 dataset (89.4%) in comparison to the P1L_S2 dataset (79.4%). This is possibly because the P1E_S2 sequences share similar traits to the P1E_S1_C1 sequence used to train the face recognizer.

**Figure 10:**
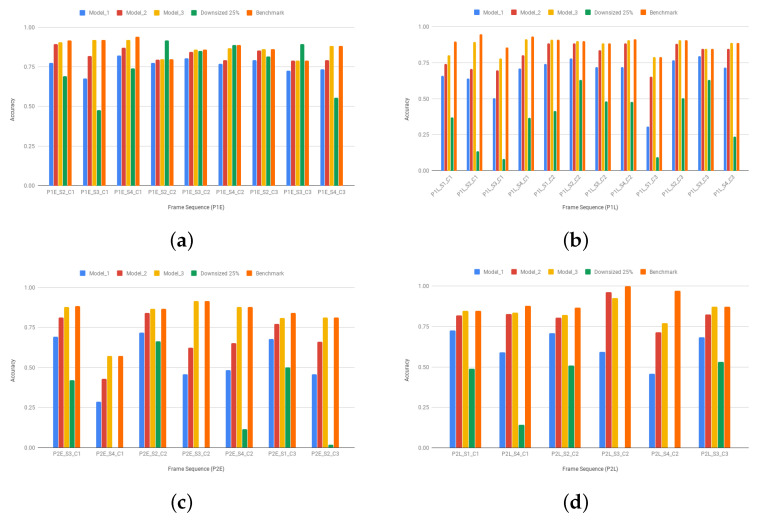
Mean face recognition accuracy regarding each evaluated model, the images resized to 25% of their original sizes, and the benchmark images from the (**a**) P1E, (**b**) P1L, (**c**) P2E, and (**d**) P2L datasets.

Figure 10 shows an expected face recognition accuracy decreasing of our ARIM-resampled frame sequences when compared to their correspondent benchmarks and to images uniformly resized to 25% of their original sizes. ARIMs rely on movement analysis to authenticate users, which creates a dependency between peripheral and the analysis of foveal information, some faces can be lost. Another variable influencing the accuracy rates is the foveal resolution of each tested ARIM. In fact, the accuracy rates increase with foveal resolution, and are not too low even under the 50% sampling degradation induced by Model_1, for example. In the case of Model_3, where foveal resolution matches that of the benchmark, the small loss in accuracy is justified by the quality of optical flow analysis, which seems to be acceptable for the tested application. Table 3 presents the minimum, mean, and maximum accuracy loss rates that are induced by each model in comparison to the benchmarks. Whereas the maximum obtained loss was 50% for Model_1 and the P2E dataset, very small loss rates (close to 0%) were registered in more than one scenario. Another interesting phenomenon is the high loss rates observed for the P2E and P2L datasets, possibly due to slight divergent conditions relative to the P1E and P1L datasets.

The accuracy results on resized images are often lower than those of models, showing a constant behavior on the P1L, P2E, and P2L datasets. However, in the P1E dataset, the accuracy for resized images were unexpectedly high in some cases. We believe this behavior may be justified by the fact that the evaluated sequences and the face training sequence share similar conditions (e.g., lighting). Additionally, the superior accuracy results of the P1E_S2_C2 and P1E_S3_C3, as compared to the benchmark, may be due to the removal of noise as a consequence of the huge resizing operation. Nevertheless, we believe these rare cases do not conflict with our general results and conclusions.

### 5.3. Energy Consumption Evaluation

**Figure 11:**
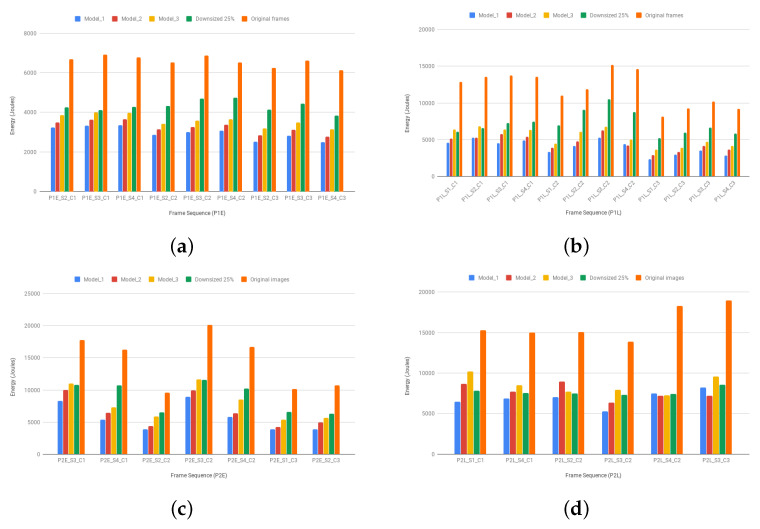
Total energy consumption regarding each evaluated model, the images resized to 25% of their original sizes, and the original (full-size) images from the (**a**) P1E, (**b**) P1L, (**c**) P2E, and (**d**) P2L datasets.

Figure 11 presents a comparison among the total energy spent by the processing of original (full-size) images, images uniformly resized to 25% of their original sizes, and the three tested models. The experiments show lower energy consumption values for scenarios that involve our models. The difference in energy values among our models and the baseline results directly from the data amount reduction caused by the combination of peripheral optical flow and the sampled foveal face detection/recognition. Therefore, the robust and timely activation/deactivation of these latter algorithms reduce the total energy spent in the whole authentication process, while keeping the accuracy rates acceptable, as previously discussed. Table 4 presents the minimum, mean, and maximum energy reduction rates that are induced by each model relative to the baseline, i.e., the obtained energy savings. As expected, the reduction rates decrease with the increase in foveal resolution, because there is more data to process. This is verifiable by a quick comparison between the mean rates of Model_1 (half density) and Model_3 (full density), for example. For resized images, the energy values are often higher than the ones produced by ARIMs, with the exception being the P2L dataset where similar values were found. This result strengthens our hypothesis that the use of ARIMs might lead to energy savings.

### 5.4. Implications in Real-Time Applications

Real-life imaging applications have a broad spectrum of possibilities, associated technologies, and challenges. These applications often need to capture and process high volumes of data—materialized in uniform images—in real-time to provide their users with the desired outputs. However, the users’ experience may be affected by these high processing demands in some degree, and should be considered during the application’s conception. On the one hand, in visual entertainment applications, users want the largest possible amount of details in pictures and videos; such a requirement can be accomplished using uniform images. On the other hand, when the final objective is related to real-time authentication, movement analysis, and action recognition, for example, non-uniform images might come into play to provide an interesting balance among accuracy, storage resources, and energy, thus possibly favoring the users’ experience.

In our simulations, the combination of uniform and non-uniform areas induced by our ARIMs allowed significantly fewer data to be processed. Besides, we noticed that the use of different pixel representations may be a good strategy in certain situations, because peripheral data may be inherently related to the application’s domain. These data may be exploited to decide when to process denser data volumes. This modeling itself already provided a significant reduction in data processing, thereby contributing to real-time performance.

Furthermore, if a hardware layer that is dedicated to extract and resample conventional images is available (e.g., implemented into an FPGA), real-time pre-processing constraints may be balanced, maintained, or even reduced, if necessary. In this scenario, the time to process frames, data volumes, energy, and computational processing power could be relieved, because many operations would be performed previously by hardware routines.

## 6. Conclusions

A crucial observation that led to the present study is that image data captured by uniform sensors are often dense and redundant, leading to computationally expensive solutions in terms of storage, processing, and energy consumption. We addressed this issue by exploiting a space-variant scheme that was inspired by mechanisms of biological vision, in particular, the way that humans sense through the retina. We introduced a generic framework for designing application-oriented retinal image models (ARIM). The models should be used to re-sample the input images prior to executing an specific CV task. We selected a biometric application to illustrate the conception and usefulness of appropriate models.

The experiments with three ARIMs having different point configurations demonstrate the flexibility of the proposed framework in devising models with different properties regarding storage requirements, energy consumption, and accuracy performance. We could observe, for example, that the use of different space-variant strategies may lead to a big reduction in terms of storage resources and energy consumption, whereas the accuracy loss rates were low in most cases. Such a trade-off evidences the viability of the proposed models and the conformity to our initial expectations regarding saving computational resources.

In future work, we intend to use our framework in other CV applications, such as surveillance and assembling line inspection. Another possibility is to represent the periphery of our models as super-pixel-like artifacts (voronoi cells) that could be filled with the grayscale pixel value at each cell’s central point in the original image. The analysis of degraded peripheral regions that are represented in grayscale might also be applied to the aforementioned application domains.

With respect to the hardware side, a promising avenue is the integration of our approach into an FPGA responsible for resampling uniform images according to some predefined or dynamic space-variant models. The models could be computed at the FPGA or by software, in which case an efficient communication mechanism between these layers should be implemented. To provide more flexibility, the collaborative use of different models and the use of computer graphics techniques to dynamically reshape them (as with the case of deformable surface models) are relevant investigations for further works. In these, a more complex repertoire of variables would need to be considered, including the costs of computing and adapting the models, resampling uniform images inside the FPGA, and the trade-off among accuracy, storage, and energy. Even with all of these variables in the field, we believe that such an infrastructure could still favor savings in the use of computational resources. Another idea that deserves some study is the use of our models along with alternative technologies for data storage, such as Network Attached Storage (NAS) systems. In this setup, bandwidth resources may benefit from the use of the space-variant image representation induced by our models.

## Figures and Tables

**Table 1:** Technical information regarding the implemented CV application. Only non-default parameter values are shown.

	Theoretical Reference	Library	Method	Input Parameters
Face Detection	Viola-Jones [20]	OpenCV 3.0.0	detectMultiScale	scaleFactor = 1.1
				minNeighbors = 3
Face Recognition	DNN model [21] + 1-NN	Dlib 19.16 [22]	get_face_chip_details	size = 150
				padding = 0.25
				winSize = (31, 31)
				maxLevel = 3
Optical Flow	Lukas-Kanade [23]	OpenCV 3.0.0	cvCalcOpticalFlowPyrLK	criteria.maxCount = 20;
				criteria.epsilon = 0.03
				minEigThreshold = 0.001

**Table 2:** Number of pixels and data size reduction results for the evaluated models relative to the original and uniformly resized images (to 25% of their original sizes).

	Num. of Pixels	Num. of Pixels Reduction	Bytes per Region		Total Bytes	Data Size Reduction
			FOV	PER		
Original	480,000	-	-	-	1440,000	-
Resized (25%)	120,000	75.00%	-	-	360,000	75.00%
Model_1	10,384	97.83%	30,000	768	30,768	97.86%
Model_2	22,884	95.23%	67,500	768	68,268	95.25%
Model_3	40,384	91.58%	120,000	768	120,768	91.61%

**Table 3:** Minimum, mean, and maximum accuracy loss rates induced by our ARIMs compared to the provided benchmarks.

Dataset	Accuracy Loss								
	Model 1			Model 2			Model 3		
	Min.	Mean	Max.	Min.	Mean	Max.	Min.	Mean	Max.
P1E	0.032	0.123	0.264	0	0.050	0.108	0	0.006	0.021
P1L	0.060	0.248	0.613	0	0.094	0.255	0	0.023	0.103
P2E	0.174	0.353	0.500	0.032	0.172	0.318	0	0.006	0.037
P2L	0.143	0.300	0.529	0.033	0.086	0.265	0	0.063	0.206

**Table 4:** Minimum, mean, and maximum energy reduction rates induced by our ARIMs compared to the provided benchmarks.

Dataset	Energy Reduction								
	Model 1			Model 2			Model 3		
	Min.	Mean	Max.	Min.	Mean	Max.	Min.	Mean	Max.
P1E	0.505	0.551	0.598	0.463	0.508	0.550	0.414	0.456	0.489
P1L	0.612	0.667	0.711	0.582	0.619	0.710	0.490	0.548	0.657
P2E	0.536	0.610	0.672	0.439	0.549	0.619	0.381	0.454	0.551
P2L	0.533	0.571	0.618	0.406	0.516	0.620	0.332	0.464	0.603
